# The Effect of Interlayer Delay on the Heat Accumulation, Microstructures, and Properties in Laser Hot Wire Directed Energy Deposition of Ti-6Al-4V Single-Wall

**DOI:** 10.3390/ma17133307

**Published:** 2024-07-04

**Authors:** Rajib Halder, Petrus C. Pistorius, Scott Blazanin, Rigved P. Sardey, Maria J. Quintana, Edward A. Pierson, Amit K. Verma, Peter C. Collins, Anthony D. Rollett

**Affiliations:** 1 Department of Materials Science and Engineering, Carnegie Mellon University, Pittsburgh, PA 15213, USA; rhalder@andrew.cmu.edu (R.H.); pistorius@cmu.edu (P.C.P.); rigved@cmu.edu (R.P.S.); 2 Department of Materials Science and Engineering, Iowa State University, Ames, IA 50011, USA; blazanin@iastate.edu (S.B.); mariaqh@iastate.edu (M.J.Q.); pcollins@iastate.edu (P.C.C.); 3 Lockheed Martin Space Systems Company, Littleton, CO 80127, USA; edward.a.pierson@lmco.com; 4 Lawrence Livermore National Laboratory, Livermore, CA 94551, USA; amitkumar1@llnl.gov; 5 Center for Advanced Non-Ferrous Structural Alloys, NSF Industry, University Cooperative Research Center, 2415 Eisenhower Ave., Alexandria, VA 22314, USA

**Keywords:** laser hot wire directed energy deposition (LHW-DED), interlayer delay, heat accumulation, Ti-6Al-4V (Ti64), single-wall structure, process–structure–property relationship

## Abstract

Laser hot wire directed energy deposition (LHW-DED) is a layer-by-layer additive manufacturing technique that permits the fabrication of large-scale Ti-6Al-4V (Ti64) components with a high deposition rate and has gained traction in the aerospace sector in recent years. However, one of the major challenges in LHW-DED Ti64 is heat accumulation, which affects the part quality, microstructure, and properties of as-built specimens. These issues require a comprehensive understanding of the layerwise heat-accumulation-driven process–structure–property relationship in as-deposited samples. In this study, a systematic investigation was performed by fabricating three Ti-6Al-4V single-wall specimens with distinct interlayer delays, i.e., 0, 120, and 300 s. The real-time acquisition of high-fidelity thermal data and high-resolution melt pool images were utilized to demonstrate a direct correlation between layerwise heat accumulation and melt pool dimensions. The results revealed that the maximum heat buildup temperature of the topmost layer decreased from 660 °C to 263 °C with an increase to a 300 s interlayer delay, allowing for better control of the melt pool dimensions, which then resulted in improved part accuracy. Furthermore, the investigation of the location-specific composition, microstructure, and mechanical properties demonstrated that heat buildup resulted in the coarsening of microstructures and, consequently, the reduction of micro-hardness with increasing height. Extending the delay by 120 s resulted in a 5% improvement in the mechanical properties, including an increase in the yield strength from 817 MPa to 859 MPa and the ultimate tensile strength from 914 MPa to 959 MPa. Cooling rates estimated at 900 °C using a one-dimensional thermal model based on a numerical method allowed us to establish the process–structure–property relationship for the wall specimens. The study provides deeper insight into the effect of heat buildup in LHW-DED and serves as a guide for tailoring the properties of as-deposited specimens by regulating interlayer delay.

## 1. Introduction

Direct energy deposition (DED) is an advanced metal additive manufacturing technique that enables us to fabricate large-scale three-dimensional complex parts with high productivity while maintaining a “near-net shape” profile [1,2,3,4]. The feedstock materials in the DED process can be in the form of powder, such as in LPBF, or a wire. In the wire-feed DED process, a wire preheated using resistive heating as the heat source is fed into the laser spot on top of the substrate to melt and deposit materials simultaneously [5]. The fabrication of large-scale components directly from the CAD model via DED requires a layer-by-layer deposition approach, which forces each layer to experience repeated heating cycles. Thermal cycles in a layer-by-layer deposition technique, such as in DED, may result in heat accumulation, which, in turn, increases the preheat temperature of the previously deposited layer [6]. In laser hot wire direct energy deposition (LHW-DED), the high energy density coupled with a high deposition rate [7,8] further aggravates the heat buildup issue. This imparts variabilities in the melt pool dimensions of the layers, resulting in deviations from the desired geometry and intended part accuracy [9,10]. Moreover, the buildup of the preheat temperature controls the cooling rates during the deposition process, which gives rise to variations in the microstructure and mechanical properties in as-built components. The microstructural evolution during multi-pass processing is complex due to the high thermal gradient and dynamic flow in a fast-moving melt pool coupled with rapid solidification and the presence of layer-by-layer deposition. Microstructural complexity is further compounded because of multiple laser passes, generating spatially nonuniform thermal reheating cycles [11].

Many researchers have sought to optimize the process parameters and conditions so as to regulate the heat accumulation and resulting properties of as-printed parts. Denlinger et al. [12] claimed that the interpass dwell time, which has a direct bearing on heat accumulation, has a significant impact on residual stress and distortion in Ti64 components fabricated by powder-fed DED. Karpenko et al. [13] showed that AM Ti6-Al-4V samples with high residual stresses had a high stress concentration at the crack tip, leading to a high fatigue crack growth rate. Wang et al. [14] studied the effect of linear heat inputs on the mechanical properties of AISI 304L stainless steel wall components fabricated by a laser-based DED process. It was discovered that the microstructures at the top of the walls were coarser than at the bottom due to a decreased cooling rate with an increased distance from the substrate, which resulted in a lower yield and tensile strength. Keist et al. [15] identified mechanical property anisotropy in terms of location and orientation in the as-built condition for laser-based DED wall structures. Jinoop et al. [16] deployed a LAM-DED (laser additive manufacturing directed energy deposition) setup to investigate the effect of process parameters on the geometry and quality of Inconel-718 wall structures. Asala et al. [17] employed Inconel 718 to study microstructure evolution with increasing build height from the substrate while measuring the temperature history during wire-arc AM. Keist et al. [15] investigated the role of wall geometry in the mechanical properties of Ti64 structures built by laser-based DED. The shape of the wall was found to be a significant parameter for single-pass thin wall structures. Furthermore, the tensile properties varied as a function of the location within the wall structures, where the tensile strength decreased linearly with increasing height above the substrate.

Although these modeling and experimental studies provided some useful perspectives, a systematic and comprehensive investigation of the evolution of the temperature profile along the build height, resulting from heat accumulation, and their consequent impact on the evolution of microstructural and mechanical properties remains an underexplored area. Residual heat accumulation is mainly regulated by adjusting the process parameters or conditions, such as reducing the energy density or applying preheat temperatures. However, since the processing parameters in DED are coupled in complex ways, tuning one parameter can have undesirable effects on the other parameters (for example, reducing the scan speed changes the original energy density and also the wire feed rate to introduce the same volume of material during the deposition), adding further complexity in establishing a one-to-one correlation between the process parameters and the resulting heat buildup. The issue of heat accumulation is further exacerbated in thin Ti-6Al-4V wall structures commonly used as structural aerospace components due to the low thermal conductivity of 6.7 W/m.k of the alloy. So, in this study, the heat accumulation in Ti-6Al-4V single-wall structures was investigated by systematically varying the interlayer delay while keeping other process parameters constant in the LHW-DED process. Adjusting interlayer delay, a secondary process variable, does not affect the energy density used for the deposition; however, it enables one to regulate the temperature gradients during the deposition process. The present study aims to examine the impact of various interlayer delays on heat accumulation and the resulting impact on the geometry, microstructure, and mechanical characteristics of as-printed Ti64 single-track-wall structures made by the LHW-DED process. Additionally, this study demonstrates how heat accumulation challenges can be effectively addressed in order to ensure excellent part geometry, as well as to minimize the heterogeneity in microstructure and mechanical properties within as-printed wall components. To the best of the authors’ knowledge, no reports are available on this topic for LHW DED-built Ti64 single-track-wall structures.

## 2. Experimental Procedure

### 2.1. Deposition of Ti-6Al-4V Wall Structures

Three 15-layer-tall and 200 mm long Ti-6Al-4V single-track-wall components were built using a laser hot wire directed energy deposition process (LHW-DED), one of the additive manufacturing (AM) techniques. Ti-6Al-4V welding wire (according to the AMS4954K specification) of 1.6 mm in diameter was used as the feedstock material. Table 1 presents the chemical composition of the Ti-6Al-4V wire used for the deposition. The process parameters were optimized to obtain the desired melt pool width, depth, cross-sectional area, and length while considering keyhole, dripping, and lack of fusion boundaries in the power–velocity processing space for the Ti-6Al-4V material. Additionally, for fabricating thin single walls, a higher minimum melt pool width was defined to successfully build thin walls that roughly correspond to the width of the laser beam. The purpose of this was to ensure that the melt pool width is large enough to avoid having only a portion of the laser power being transmitted to the previously deposited bead, leading to a progressively skinnier wall with increasing height.

**Figure 1:**
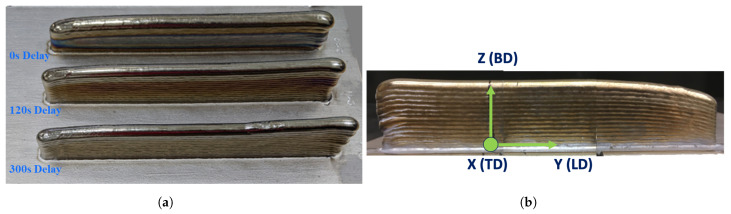
(**a**) Photographs of three wall specimens built with distinct interpass dwell time, 0, 120, and 300 s; (**b**) (not to scale) An image of the front view of a wall specimen with the axis convention highlighting the build direction, longitudinal direction, and transversal direction. The XZ and YZ planes are transversal and longitudinal cross-sections of the wall specimen.

The fabrication of wall structures entails feeding the preheated wire to the laser spot onto a Ti64 build plate (per the AMS4911R specification), while the ABB 6700 series industrial articulated robot system integrated with the laser and wire deposition functions moves in a synchronized fashion to deposit each layer. Wire preheating was accomplished using Lincoln Electric Power Wave welding technology with feedback control to monitor and suppress any sustained arcing. The deposition was carried out in a controlled, inert Argon-2 gas environment to achieve extra-low interstitial grade deposits. To control the environment, the tool head was placed within a sealed flexible PVC bag, which was coupled with an inert Argon-2 gas management system. The oxygen level was monitored throughout the deposition process using a sensor, and the ppm level was checked at the start and end of the deposition process of each wall structure. A set of optimized process parameters was used for deposition, including an input laser power of 5 kW, a hot wire power of 0.3 kW, a scan speed of 5 mm/s, and a wire feed rate of 57.5 mm/s. To investigate the effect of interlayer delay, walls were built with an interlayer time delay of 0, 120, and 300 s with optimized process variables. Figure 1 presents the photograph of the wall structures. During deposition, the dimensions of the in situ molten pool, the temperature of the melt pool, the temperature of locations that were 38 mm behind and ahead of the melt pool, and the positions of the robot arm were recorded as time series data. The Allied Vision Prosilica GT1930 camera, which was calibrated using a dimensional grid at Oak Ridge National Laboratory, was used to measure the dimensions of the melt pool. Optris CT XL 3M, Optris CTlaser 3M, and Optris CTlaser 05M pyrometers were employed to record the temperature on the surface of the previously deposited layer from positions that are trailing, leading, and within the melt pool during the deposition process. The pyrometer data were calibrated at a constant emissivity using the manufacturer’s listed accuracy values, and the measurement precision was found to be ±25 °C. Inputs acquired from individual sensors were stored in the Data Acquisition System (DAQ). The time series data were analyzed using a Python script.

All samples were prepared by sectioning the walls transverse to the scan direction to fit a 32 mm mount using the wire cut electrical discharge machining (EDM). Wall structures with heights exceeding the diameter of the mount were further sectioned to fit multiple mounts. The samples were mounted in Buehler KonductoMet phenolic powder and polished for metallographic analysis and further characterizations according to the three-step procedure described by Springer and Ahmed [18].

### 2.2. Thermal Modeling

A one-dimensional thermal model was developed based on a numerical method, approximating the continuous temperature distribution using an explicit enthalpy-based finite-difference approach. The main assumption in this model is that the temperature gradient is considered only in the build direction. This is a reasonable first approximation, given the horizontal orientation of the characteristic microstructural bands observed in these single walls (indicating the lateral temperature differences are small) and that the test builds are long (in the laser travel direction) compared with the thickness of the deposit.

At set time intervals, a defined volume of material (based on the layer thickness and deposit width) is added to the build, and a certain increment of heat is transferred to the top of the build. The additional energy is added at a constant rate to the deposited volume over a defined time period; this time period corresponds to the time it takes for the laser and heated wire to pass over this position. The temperature at each node is calculated using an inverse function to convert the volumetric enthalpy to the temperature.

**Figure 2:**
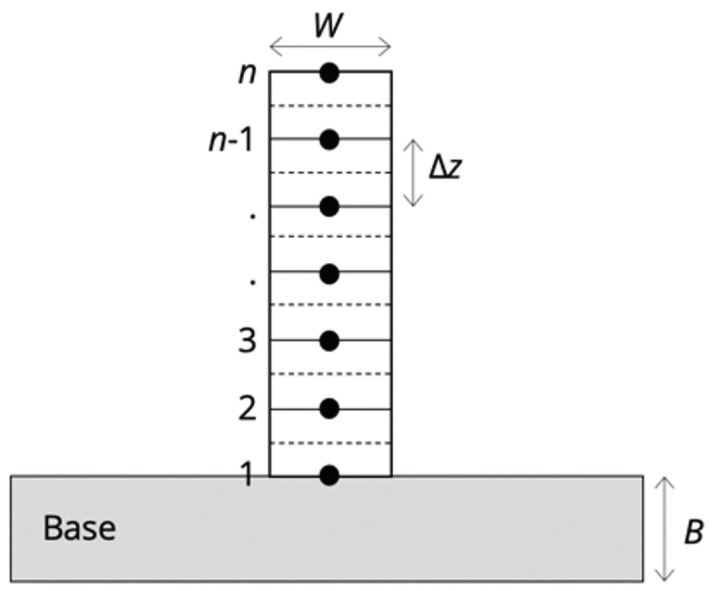
The schematic diagram illustrates the discretization of a layer used to estimate heat flow resistance in the thermal model.

For nodes 2 to *n* − 1, as shown in Figure 2, the conduction resistance in both the positive and negative z directions was calculated by the expression as follows:

(1) $$Resistance_{cond}=\frac{\Delta z}{k\cdot W}$$

where *k* is the thermal conductivity evaluated at the average temperature, *W* is the width of the wall, and Δz is the node thickness. For node 1, the resistance in the positive *z* direction was estimated using Equation (1). The resistance in the negative *z* direction was estimated based on the pseudo-steady-state conduction into the base plate by the shape factor [19]:

(2) $$Resistance_{substrate}=\frac{{\left\{\log\left[(B+W)/W\right]\right\}}^{0.59}}{1.45\cdot k}$$

where *B* is the thickness of the base plate. Radiation and convection resistances were described by a joint heat transfer coefficient:

(3) $$h_{total}=h_{conv}+h_{rad}=h_{conv}+\sigma \cdot \epsilon \cdot \left(T_{s}^{2}+T_{\infty }^{2}\right)\cdot \left(T_{s}+T_{\infty }\right)$$

where $T_{s}$ is the node temperature, $T_{\infty }$ is the temperature of the surroundings, ϵ is the emissivity, and σ is the Stefan–Boltzmann constant. The emissivity for the absorption of the laser wavelength was initially taken to be 0.15, as used by Kelly and Kampe [20] but adjusted to match the observed white-band spacing as mentioned later, and the emissivity for radiative transfer was taken to be 0.4 [21]. For nodes 2 to *n* − 1, the resistance to the surroundings was 1/2hΔz, whereas for nodes 1 and *n*, the resistance was taken to be 1/hΔz and 1/h(Δz+W), respectively.

The thermophysical material properties used in the model were taken from the compilation of Mills [22]. The thermal conductivity of the liquid alloy was taken to be 15 times the liquid conductivity at the liquidus temperature, to account for the convection of the liquid metal. A grid sensitivity test was performed to compare the temperature histories with different node heights for calibration. A standard node height of 0.5 mm was chosen based on the grid sensitivity test results. Moreover, the model’s precision was validated by contrasting the projected positions of the white bands as predicted by the model with the positions measured from the wall structures. The model takes process parameters, interlayer delays, bead dimensions, base plate thickness, emissivity, and the natural convection heat transfer coefficient as input to predict the temperature profile for the layers in the wall structures.

### 2.3. Wall Geometry and Microstructure Quantification

The dimensions of the wall structures were measured from their optical images using ImageJ, an open-source image analysis software [23]. Optical microscopy was performed using a Zeiss LSM 800 confocal microscope. The sample preparation involved cutting and sectioning the build plate using electrical discharge machining (EDM) to fit it into a 32 mm mount. All samples were mounted in Buehler KonductoMet Phenolic powder and subsequently polished using a three-step polishing method adapted from [18]. Following polishing, samples were etched by submerging them into Kroll’s Reagent for 15 s to capture microstructural features under an optical microscope. Subsequently, ImageJ was used to extract the bead features using OM images.

To quantify the microstructural features, such as the alpha lath spacing and volume fraction of phases, and to capture their variation along the wall height, a series of SEM images was taken starting from the base to the top of the wall following a vertical line positioned at the center of the wall structures. All images were recorded at 5000× with an accelerating voltage of 15 kV in the backscattered (BSE) imaging mode. The magnification of 5 KX provided a reasonable pixel resolution and sufficient features to count. A 15 kV energy ensured sufficient contrast in the BSE images without flooding or blurring them by sampling more subsurface features.

An open-source software package, MIPar [24], was used to measure alpha lath thickness and the volume fractions of phases. The MIPar software package permitted for image noise reduction, brightness and contrast adjustment, and image segmentation. The alpha lath thickness was determined by measuring the average feature length using 1000 lines drawn at a random orientation and random location on the image and then plugging the average feature length into Equation (4) [25]. The feature length, λ, is defined as the length of the line occurring within two beta ribs that are seen as thin lines in the BSE images.

(4) $$Lath\;Thickness=\frac{1}{1.5\cdot {\left(\frac{1}{\lambda }\right)}_{mean}}$$

### 2.4. Compositional Analysis

The chemical composition of the wall structure was measured using an FEI Quanta 600 FEG SEM intrument, which is equipped with an analytical system and an XMAX 80 mm SDD EDX detector. The whole system was connected to a support PC that has Oxford INCA EDS chemical analysis software. EDS spectra were collected at a working distance of 10 mm with electron accelerating voltages of 12–15 keV. The output of an EDS analysis was a plot of X-ray counts vs. characteristic energy in electron volts (eV). The precision of the EDS measurements was ensured by allowing the acquisition to continue until 50,000 counts were collected in the spectrum for quantification from a mirror-finished sample surface. The Oxford software package was used to quantify the concentrations of Al, Ti, and V by weight for each spectrum collected. CASINO, a Monte Carlo simulation program (https://www.gegi.usherbrooke.ca/casino/What.html, accessed on 11 April 2023) that models electron beam–sample interactions, was used to estimate the interaction volume in the bulk of the Ti-6Al-4V wall structure to ensure that the volume was large enough to capture the composition from both the alpha and beta phases and, thus, represents the overall composition of the bulk of wall components.

### 2.5. Mechanical Testing

#### 2.5.1. Vickers Hardness

An automated Vickers hardness test machine from LECO AMH55 was used to test the hardness of the as-printed Ti64 parts. A load of 1 kg-force, spacing of 500 μm between indents, and loading time of 15 s were used for the test. The software integrated with the hardness machine automatically reports the HV number by calculating the ratio of F/A, where F is the force applied in kg-force and A is the surface area of the resulting indentation in square millimeters [26].

#### 2.5.2. Tensile Testing

Eighteen tensile test coupons were excised via EDM from the single-bead walls that were made with 0, 120, and 300 s interlayer delays. The number of coupons tested from each wall was 6. Each tensile specimen was tested according to the ASTM E8–15a standard and at a strain rate of 0.005 min${\;}^{-1}$ (8.3 × 10${\;}^{-5}$ s${\;}^{-1}$), with a strain rate jump to values of 0.05 min${\;}^{-1}$ (8.3 × 10${\;}^{-4}$ s${\;}^{-1}$) after the onset of yielding.

## 3. Results

### 3.1. Thermal History

**Figure 3:**
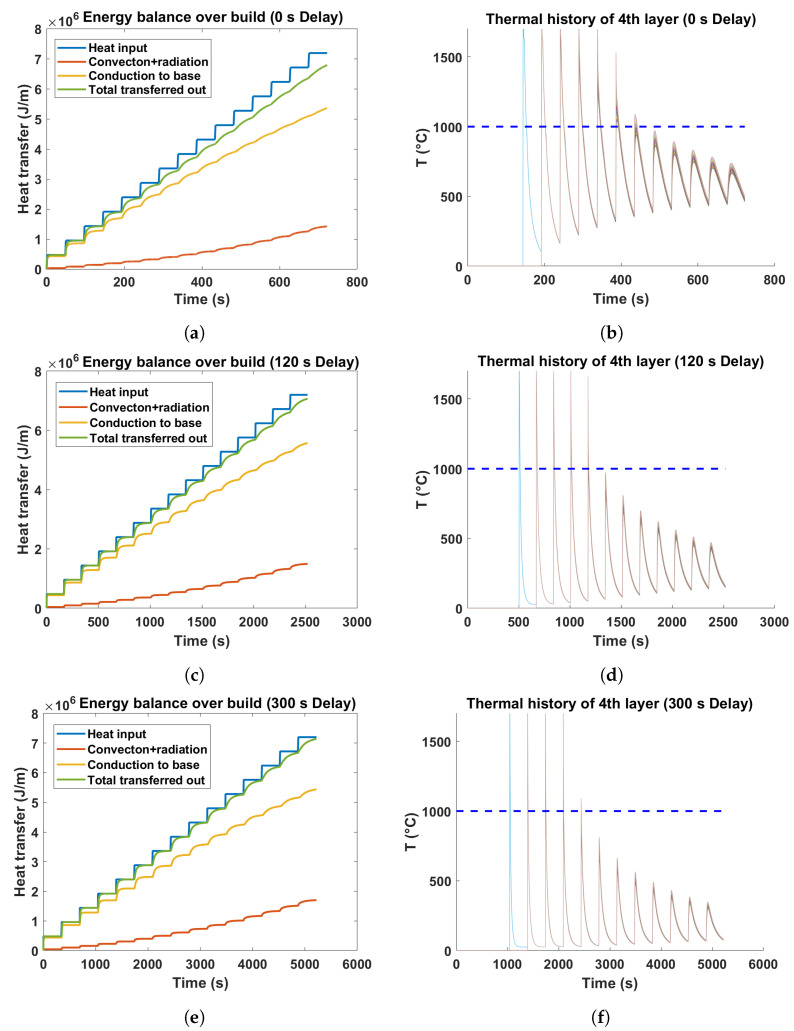
(**a**,**c**,**e**) The plots showing energy balance over the build for three distinct interpass dwell time. The total energy input, the dissipated energy, and the contributions of different modes of heat transfer to the energy transferred out are highlighted. (**b**,**d**,**f**) Thermal histories of the fourth layer in wall specimens are illustrated for three interlayer delay conditions. The horizontal broken line at 1000 °C indicates the β-transus temperature of Ti-6Al-4V used in the thermal model.

Figure 3 illustrates the energy balance over the build for three wall structures deposited with different interlayer delays. The flat segments within the heat input curve represent the time lapse between subsequent deposits at a given position , which is computed by dividing the wall length of 200 mm by the scan speed (5 mm/s) and subsequently incorporating the interlayer delay as well as the time for the robot to return to the starting position. In contrast, the vertical line represents the time interval over which the heat is transferred for each deposited layer, determined by the ratio of the beam width to the laser speed. The gap between two curves representing the heat input and total transferred out in Figure 3a,c,e implies heat accumulation over the course of the deposition. The model predictions suggested that the wall with a 0 s delay would experience higher heat buildup as compared to walls made with an extended delay. This can be inferred from the relatively larger difference observed between the total heat input and output in the case of a 0 s interpass dwell time. Moreover, the model implied that the issues of heat buildup would be more pronounced at the upper portions of the wall compared to its base, as the gap between total heat input and output progressively increases with increasing wall height. Furthermore, the increment of valley temperatures for the fourth layer of the wall with 0 s delay, shown in Figure 3b, signifies heat accumulation over the build. This gradual increase in valley temperatures is minimized by the introduction of an extended delay between the deposition of successive layers, as demonstrated in Figure 3d,f. In addition, the thermal model delineated that the principal mechanism of heat transfer is conduction through the base plate, while the layers positioned farther above the build plate see an increasing importance of heat dissipation mechanisms involving radiation and convection.

The temperature histories of individual layers were also estimated for three distinct delays. The progressive increment of valleys in the temperature curves in the instance of the briefest delay suggests that the preheat temperature continuously increases during the deposition process, in contrast to the pattern observed in the case of a 300 s delay. It is also evident from the plots that a specific layer within the wall experiences a different thermal history due to the heat accumulation issue resulting from the imposed interlayer delay—essentially forced cooling between the deposition of successive layers. The steep slope of the cooling curves of the individual layers for a 300 s delay also implies higher cooling rates due to the presence of a high thermal gradient. Therefore, interlayer delay, i.e., an enforced cooling, emerges as an adjustable process parameter capable of governing the preheat temperature of layers within the wire-feed DED process.

### 3.2. Heat Accumulation and Melt Pool Dimensions

**Figure 4:**
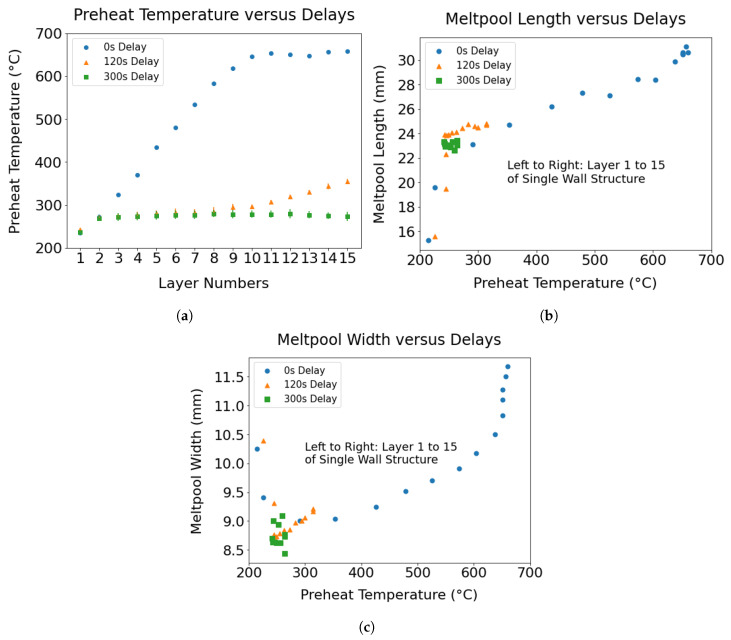
(**a**) The effect of interlayer delay on heat accumulation is demonstrated by plotting the evolution of preheat temperature of individual layers in the wall as a function of delay; (**b**,**c**) The evolution of the melt pool length and width over the deposition is highlighted by capturing the variation in the melt pool dimensions of individual layers during the deposition of the wall specimens made with distinct interlayer delays.

The melt pool dimensions of each layer were derived from in situ time series data, which were recorded during the deposition of the wall components. The in situ melt pool dimensions recorded in pixels were converted further to millimeters using a conversion factor of 8.81 pixels/mm. The data allowed us to capture the evolution of the melt pool dimensions, as well as the preheat temperature by deriving the information from individual layers. A continuous increase in the preheat temperature of a previously deposited layer while a new layer is to be laid on top of it reflects heat accumulation. The temperature of the previous layer while printing a new layer was measured using a pyrometer located 38 mm ahead of the melt pool at the time of deposition. To elucidate the influence of heat buildup on melt pool dimensions, the mean width and length of the melt pools, as well as the preheat temperature for each layer are calculated and graphically represented in Figure 4.

**Figure 5:**
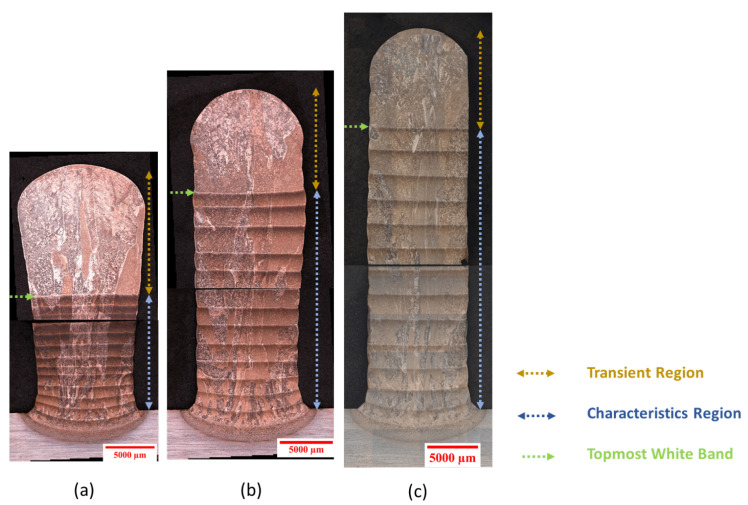
Optical micrographs (OM) of the cross-sections of the wall specimens. (**a**–**c**) Three wall specimens fabricated with distinct interpass dwell time, i.e., 0, 120, and 300 s, respectively. The arrows annotated on the images highlight the wall features such as the characteristic region, transient region, and topmost white band. There is a total of 14 white bands in each wall. The scalloping of the wall with height is evident for the scenario of the shortest delay from the OM (**a**).

The interlayer delay has a direct effect on heat buildup, resulting in a variation in the dimensions of the melt pool, as can be seen in Figure 4. An increase in interlayer delay limited the heat buildup and thereby provided increased stability of the melt pool dimensions. On the contrary, a continuous increase in heat accumulation was observed in the case of the shortest delay. Moreover, an approximately linear relationship was found to exist between heat accumulation and ensuing changes in the melt pool dimensions. Variabilities in melt pool dimensions due to heat buildup resulted in poor dimensional accuracy of the final parts, as is evident from the shape of the single wall built without interlayer delay (Figure 5a). Upon comparing the melt pool dimensions of the topmost layer under 0 and 300 s delays, a rise of approximately 32.9% in the melt pool width and around 32.7% in the melt pool length was evident in the instances of the shortest delay, attributed to heat accumulation, relative to the case of a 300 s delay. The preheat temperature for the topmost layer reduces from 660 °C to 263 °C with an increase to a 300 s delay.

### 3.3. Characteristics of the Wall

Figure 5 shows a montage of an optical micrograph of the cross-section of the wall structures fabricated maintaining different delays between layers during deposition. The typical characteristic region, transient region, and white bands are highlighted in the optical images. These features are defined as follows: the parallel and horizontally oriented lighter features observed in the cross-sectional image of a wall structure under the optical microscope are called white bands. The white bands and their spacing are related to thermal phenomena, and the mechanism of the formation of white bands is explained in Section 4.1. The characteristic region is defined as the area enclosed by the base plate and the topmost white band. In this characteristic zone, a repeated pattern of a white band followed by a darker region is observed. The location of the white bands is not associated with the layer positions as such. The region located above the topmost white band is called the transient region. The individual beads can be distinguished by the curvature present at the edges of the cross-sectional images of the wall components. The coarsening of the microstructure can be clearly identified from the bottom to the top, with much smaller grain sizes on the base plate. The grains are several millimeters wide in the horizontal direction and elongated in the build direction, which agrees with the epitaxial growth mechanism under conditions of vertical heat flow. Similar results were found in previous investigations with similar processing conditions [21,27,28].

Different interlayer delays applied between these wall structures, resulting in different preheat temperatures of the previously built layer, govern the shape of the wall, the dimensions of the wall, and the location of the white bands in the wall structures. It can be seen in Figure 5 that the wall built without delay assumes a wedge shape where the bottom of the wall is narrower than its top. The accumulation of heat with increasing deposition height causes the size of the melt pool to increase over the course of the build, which then results in a progressive growth in the lateral dimensions of the part [11]. The other two walls printed with a relatively longer delay possess a uniform width along the wall height, which is in line with a lower heat buildup during deposition. The uniform wall width with an increase in interpass delay is mainly due to the reduction in the lateral expansion of the melt pool resulting from the reduction in the preheat temperature. The driving force behind the fluid flow within the melt pool is due to the Marangoni effect or the thermocapillary effect, in which fluid motion is induced by changes in surface tension with temperature [29]. The area of the transient region was found to be larger for the wall with a 0 s delay.

**Figure 6:**
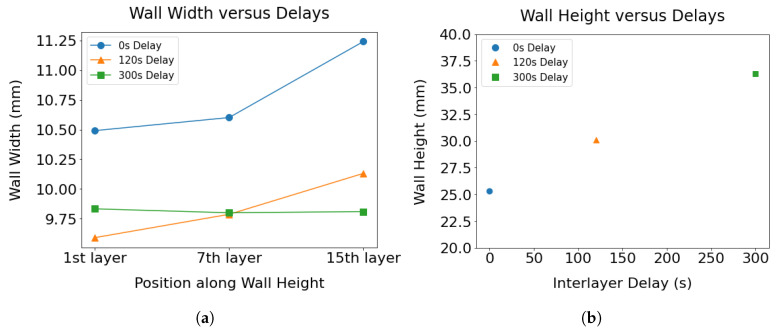
(**a**) Variation of wall width across the height captured for walls as a function of interlayer delays. (**b**) The height of the three walls with different delays measured from the deposition end point are compared to demonstrate the effect of heat buildup on the wall dimensions.

An open-source software, ImageJ [23], was used to measure the width and height of the wall structures to capture the variation in the bead dimensions due to heat accumulation. The width was measured from three distinct positions of the wall structures—(a) at the base of the wall at a height of the second layer above the build plate, (b) at the center of the wall, and (c) at the top of the wall before the outer edges of the wall start to curve. The height of the wall represents the distance between the top of the wall and the surface of the base plate. The role of the interlayer delay on the dimensions of the beads is illustrated in Figure 6. Figure 5a demonstrates that the bead width progressively increases with increasing wall height when the shortest delay is maintained between the deposition of layers. In contrast, with interpass delays of 120 and 300 s, a consistent bead width is observed throughout the entire wall height. An increase in 40% wall height was observed with an increase in delay of 300 s. It is worth noting that the wall height is not uniform throughout the length of the wall because a slumping behavior was observed at the deposition end point. Hence, depending on the sampling location, the measured wall height would differ. The heights of the three distinct walls reported in Figure 6b were from the samples taken at the same location from these walls.

### 3.4. Compositional Variations

**Figure 7:**
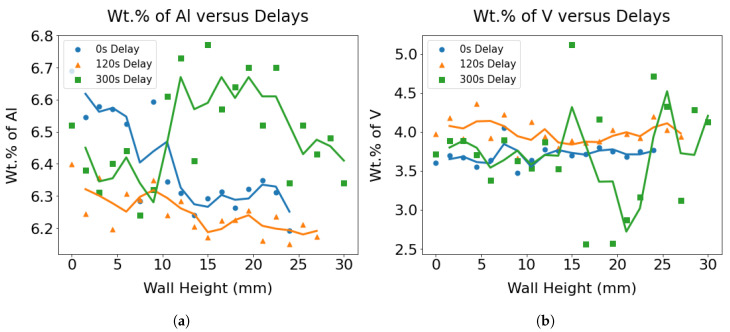
The changes in composition are captured via the variation of (**a**) wt.% of Al and (**b**) wt.% of V across the wall height for three wall specimens with different delays.

The mapping of the chemical composition was performed by energy dispersive spectroscopy (EDS). Composition data were collected from various positions along the height of the wall, maintaining a 300–500 μm spacing between them. Then, an average of 3–4 data points with a progressive height increase was taken to elucidate the variations in composition with height. A decreasing trend of Al wt.% was recorded with an increase in the build height for the wall made without interlayer delay, as shown in Figure 7a. This can be attributed to a considerable elevation in the temperature of the melt pool due to the absence of interlayer delay, resulting in notable preferential vaporization effects for aluminum relative to other elements [30]. Zhang et al. also [31] reported that, when the laser energy density exceeds 126 J/mm^3^ in the Ti-6Al-4V SLM process, the temperature of the molten pool is significantly elevated to contribute to a remarkable vaporization loss (>0.15%) of Al. According to the ASTM B381-13 standard specification for titanium and titanium alloy forgings [32], changes in the mass fraction of the Al element in formed components greater than 0.15% are considered unacceptable. Similarly, a weak decreasing trend in the Al content with substantial scatter was apparent for the sample with delays of 120 and 300 s. Figure 7b demonstrates changes in the wt.% of V with wall height as a function of the delay. No discernible trend of the variation in %V was found along the build height.

### 3.5. Microstructures

**Figure 8:**
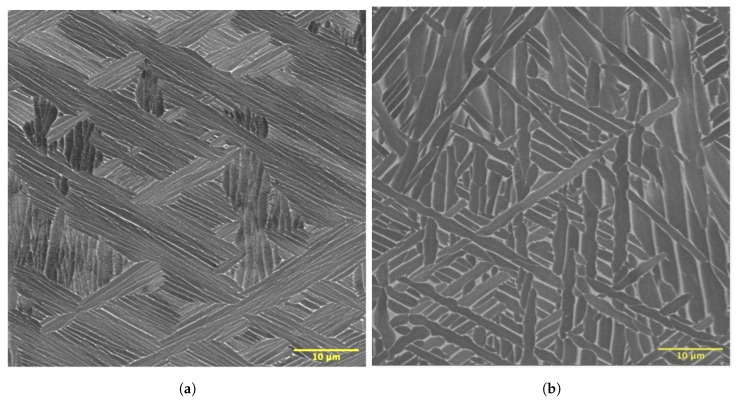
(**a**,**b**) BS SEM images of typical colony and basket weave microstructures observed in the wall specimens.

The microstructural features of as-built wall structures were investigated using scanning electron microscopy (SEM). Typical backscattered SEM images of the colony type and basket weave microstructures consisting of alpha and beta phases are shown in Figure 8a and Figure 8b, respectively.

**Figure 9:**
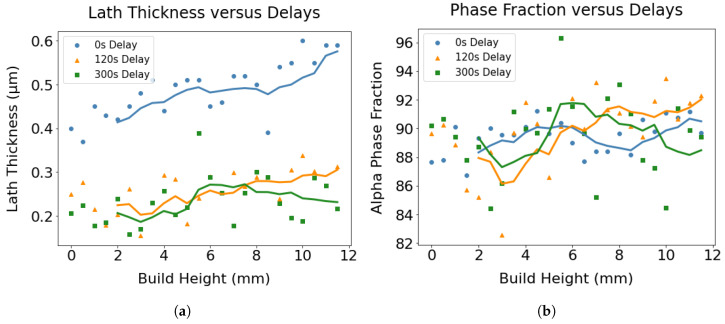
The variation of (**a**) lath spacings and (**b**) phase fractions of three walls with distinct delays.

When lath thickness values were plotted as a function of build height up to 15 mm considering only the characteristic regions (Figure 9a), an increasing trend of lath thickness with increasing build height was observed for all three samples. The degree of coarsening of lath thickness was more prominent for the wall made without delay, which can be ascribed to the decelerated cooling rates caused by the accumulation of heat. Moreover, the shortest delay resulted in a higher average lath thickness, suggesting relatively coarser microstructures. This is because the layers experience consistently higher temperatures throughout the build duration without delay, in contrast to when a prolonged delay is implemented. For example, the estimated temperature profiles of the fourth layer for 0 s, 120 s and 300 s delays depicted in Figure 3b,d,f demonstrate that the overall temperature of the 4th layer for the wall without dwell time sustained higher temperatures during the deposition process compared to walls with extended delays.

The volume fractions of the phases were found to be approximately 90% alpha phase and 10% beta phase. Figure 9b shows the change in the volume fraction of the alpha phase with increasing height for the three wall structures. No noticeable pattern in the phase fraction was observed in relation to the height of the wall.

SEM images taken within the transient region confirmed that the $\alpha_{colony}$-type microstructure was prevalent in it. This is due to slower cooling rates, that is 15–35 °C/s in the transient region, which allows enough time for the alpha phases to orient themselves in the same direction and grow in a parallel fashion. The characteristic regions were comprised of a mixture of the colony- and basket weave-type microstructures. The grain sizes observed in the transient region were much coarser than those of the characteristic region.

### 3.6. Vickers Microhardness

**Figure 10:**
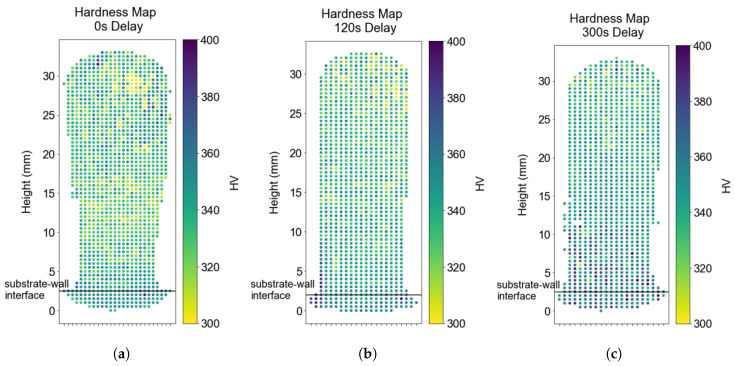
The hardness maps of three walls with distinct delays depicting the location-specific hardness variation in the wall specimens. (**a**) Hardness map—0 s Delay. (**b**) Hardness map—120 s Delay. (**c**) Hardness map—300 s Delay.

Vickers microhardness mappings were acquired to quantify the variation in the mechanical properties within the wall components, correlating with interlayer delay. The hardness maps in Figure 10 highlight the variation in hardness along the build height on these wall structures. This variation can be attributed to the coarsening of the lath thickness along the build direction, as shown in Figure 9. The hardness map with a longer delay appears to have uniform hardness values along the wall height owing to the (effectively) uniform cooling rates as a function of height.

**Figure 11:**
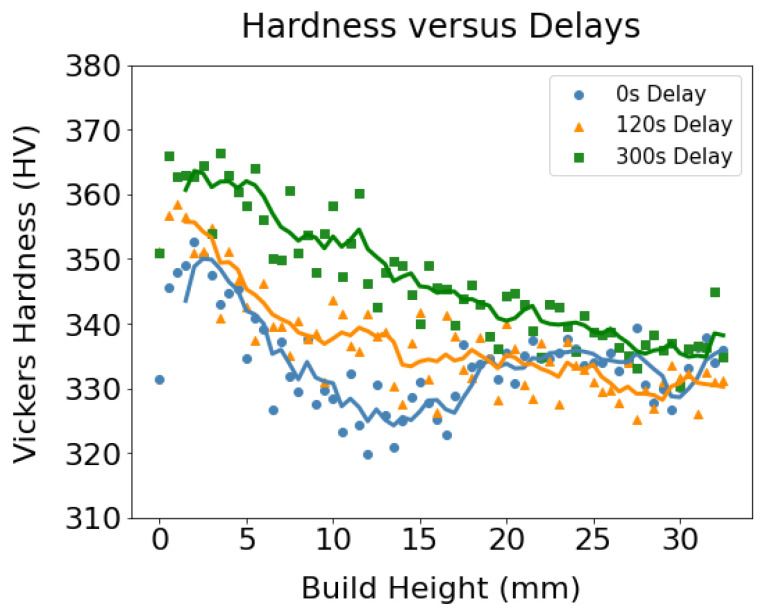
The hardness variation is captured with increasing wall height for the three walls with distinct interpass dwell time.

To capture the variation in hardness along the wall height, Figure 11 was generated by taking the average along an array of horizontal indents and then repeating the process for the next vertical position. All three samples showed a decreasing trend of hardness with increasing wall height.

**Figure 12:**
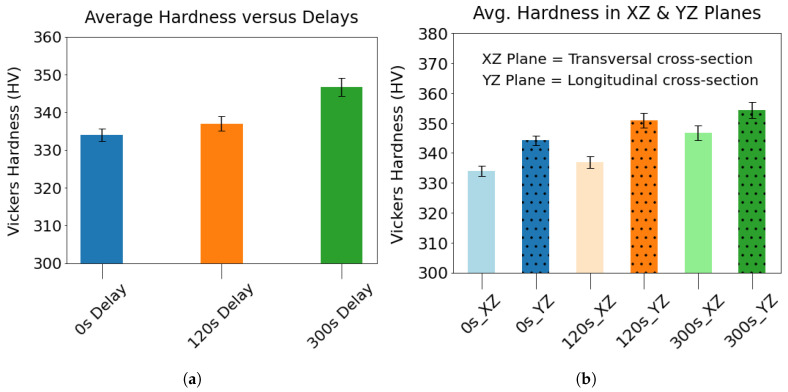
(**a**) The changes in the average Vickers hardness is plotted with respect to the interlayer delays. (**b**) The comparison of the average Vickers hardness between the transversal and longitudinal cross-sections of the three walls. The error bars represent the 95% confidence interval of the hardness.

Figure 12a highlights that a longer delay leads to a higher average hardness in these Ti64 wall components. Approximately a 4% increase in average hardness was observed when the interlayer delay increased from 0 to 300 s for these Ti64 wall components. The anisotropic mechanical behavior of Ti64 was captured by measuring the hardness in the XZ and YZ planes of the walls (Figure 12b). The hardness of the YZ planes was found to be higher than that of the XZ planes for all three samples. This observation is in agreement with the existing literature [33,34,35]. The hardness variation in different cross-sectional planes can be explained by the crystallographic orientation of the underlying α phase in them. In an experimental study, Viswanathan et al. [36] found that the hardness obtained by nanoindentation on the α grains presenting the basal plane exhibited 50% higher hardness than the α grains presenting the prism planes.

### 3.7. Tensile Properties

**Figure 13:**
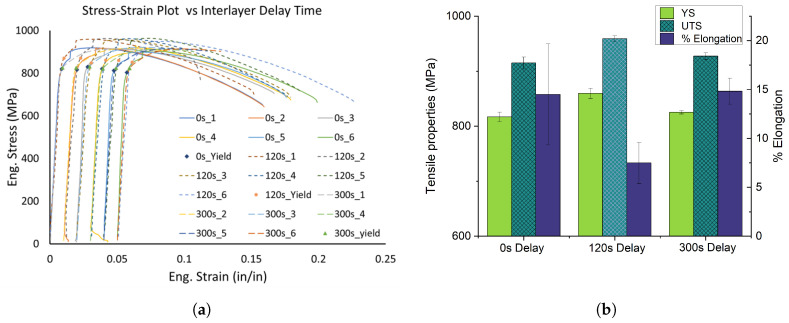
(**a**) The stress–strain plots of the three wall samples with distinct interlayer delays. A total of six samples were tested for each delay, and the tensile curves are plotted with an offset of 1% strain for visual clarity. (**b**) The bar plots illustrating the tensile properties as a function of interpass dwell time.

The tensile tests were conducted on six specimens of each of the three wall samples. Slightly different testing practices and protocols were used for samples with 300 s delay where the extensometer was removed after the onset of yielding. So, the strain data were not collected after about 1.2% strain, however, the load was captured until breaking. To ensure that the results are comparable, the modeling approach based on a Kocks–Mecking–Estrin formulation was used to predict the plastic behavior of the 300 s delay specimens after the removal of extensometers. However, this modeling is only valid until the ultimate tensile strength, and the 300 s delay specimens exhibited greater ductility than shown in Figure 13a. Both the 300 s data, as well as the raw strain data for the 0 and 120 s delay specimens (six specimens each) are plotted in a single graph, with a strain offset of 0.01 incorporated to help visualize the data (Figure 13a). In general, samples with an extended delay showed improved yield strength as well as ultimate tensile strength. Table 2 summarizes the tensile test properties of the three wall components. Although a reduced % elongation was observed at break for samples with 120 s delays, the 300 s samples had nominally the same elongation, suggesting that a typical trade-off between strength and ductility can be pursued with an application of extended delays in the fabrication of the wall sample, but that additional work would be needed to optimize such a delay. The tensile properties observed in this study align with the findings reported in the literature, as shown in Table 3 [37,38,39,40,41,42,43,44,45,46].

**Figure 14:**
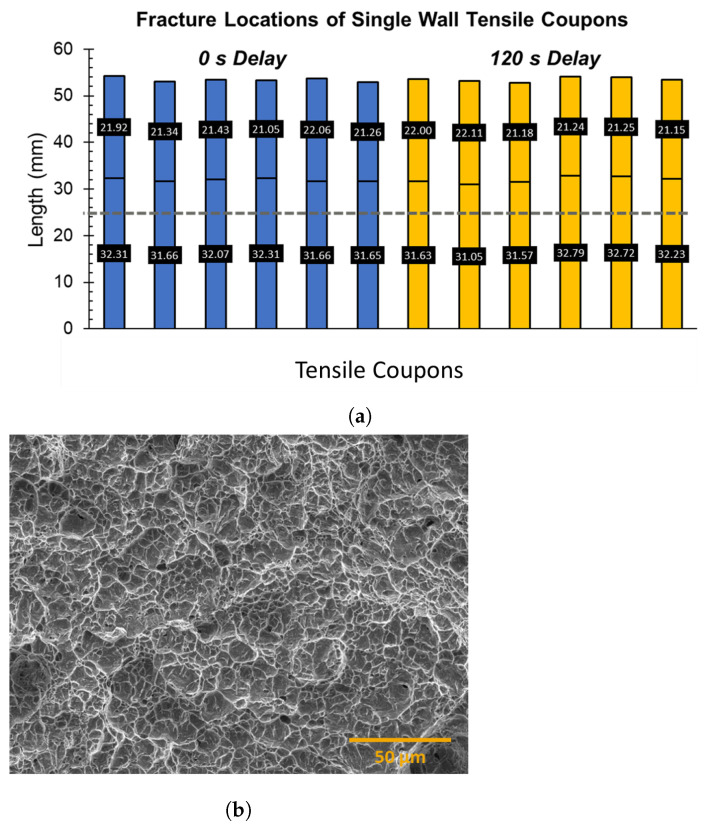
(**a**) The bar plots showing fracture locations of tensile coupons tested from single-wall structures made with 0 and 120 s delays. The grey dotted line represents the wall–substrate interface, whereas the solid black line indicates the fracture location in the tensile coupon. The length of the broken halves of the tensile coupon is also highlighted in the bar plots. (**b**) SEM micrograph of a fracture surface of the tensile coupon illustrating the ductile failure mechanism for these single-wall tensile specimens.

The failure analysis of the tensile coupons revealed that the fracture locations in the tensile coupons of these single walls were approximately 8–9 mm above the wall–substrate interface and were located mainly in the fourth layer of the wall, as shown in Figure 14a. The interface and the fracture locations are represented by the gray dotted line and the solid black line, respectively, on the bar plots. The length of the broken halves of the tensile coupon is also highlighted in the plot. The variation in the location of the fracture was found to be low, with a range of 1.10 and 0.96 mm for delays of 0 and 120 s, respectively. The location of the fracture site could be related to the reduced wall strength in that location, which could be significantly lower than the strength of the substrate. The cooling rates drop sharply to low values at a height of ≈10 mm in the wall, resulting in the reduction of the Vickers hardness by 20–25 HV at that height of the wall relative to the interface. We hypothesize that this reduction in strength at a height of ≈10 mm from the wall–base interface makes the region significantly softer than the substrate, allowing the initiation of a necking in that region, which eventually leads to failure. The fractography of the tensile coupons illustrated in Figure 14b confirms the development of ductile fracture in the tensile coupons that starts with a localized necking and ends with the rupture of the tensile coupons.

## 4. Discussion

### 4.1. Formation of White Bands

During repeated heating and cooling cycles in the deposition process of Ti-6Al-4V, a white band forms at the location where the temperature reaches within ≈30 kelvin below the transus, as proposed by Ho et al. [47]. It is assumed that the band forms if the last temperature excursion above ≈975 °C does not cross the β transus. As can be seen in Figure 5, the white bands appear slightly brighter than other locations in the characteristic region, because they predominantly consist of fine lamellar and colony-type microstructures. As argued by Ho et al. [47], when the temperature reaches close to the transus upon heating, a complete transformation of primary α does not occur, and consequently, a significant amount of α with lamellar structure is retained, which then provides additional growth sites for α to grow in parallel during cooling. This growth mechanism increases the interfacial area of the α and β phases per unit volume. Because the lamellar structure more effectively scatters the light in the same direction than randomly oriented microstructures, it appears brighter on the optical micrograph.

**Figure 15:**
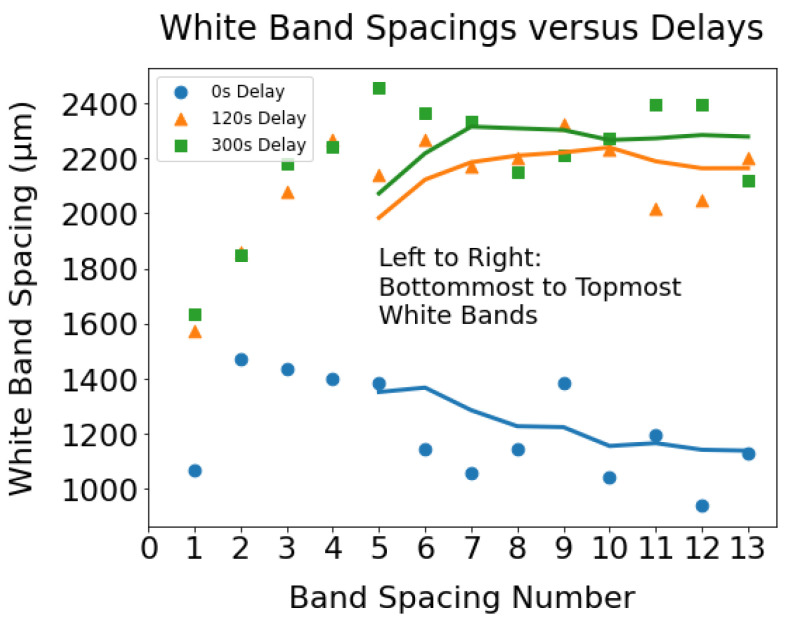
The white band spacings in the walls with distinct delays demonstrating that the white bands closely spaced in the wall with the shortest interlayer delay. The solid lines representing the moving average of white band spacings illustrate that the gaps between bands progressively reduce with increasing wall height for 0 s delay.

In this study, a total of 14 white bands were observed in the 15-layer tall wall specimens. However, the spacings between bands were different for cases with different interlayer delays, as shown in Figure 15. Figure 15 illustrates that the bands are much more closely spaced for the wall made without any delay than that of other walls. These microstructural features can be attributed to a higher heat buildup observed in the case of a 0 s interlayer delay. As heat accumulation increases the preheat temperature of the last deposited layers, when a fresh layer is deposited on top of the previously built layer, the location in the fresh layer that reaches a temperature within ≈30 Kelvin below the transus resides close to the white band formed in the last layer. Higher remelting of the previously built layer due to its higher preheat temperature resulting from increasing heat accumulation compresses the white bands together and forces them to be tightly spaced as the build progresses. The continuous decrease in band spacing seen in Figure 15 for the wall made without delay indicates an increasing trend of heat buildup as the build progressed.

In the optical images (Figure 5) of the wall structures, bands closer to the base plate were found to have a curvature compared to bands positioned away from the base plate. This phenomenon can be explained by the heat dissipation mechanisms of the wall structure. Heat flow is two-dimensional for layers that reside closer to the base plate. Heat extraction occurs via conduction into the build plate, where the heat is removed vertically in the downward direction from the center of the wall and in the sidewise direction from the edges of the wall. On the other hand, the heat flow becomes increasingly one-dimensional for layers away from the base plate as the heat is removed through previously deposited layers via conduction. This process of heat flow gives rise to the flat and horizontal orientation of the microstructural bands, indicating that the lateral temperature differences are marginal.

**Figure 16:**
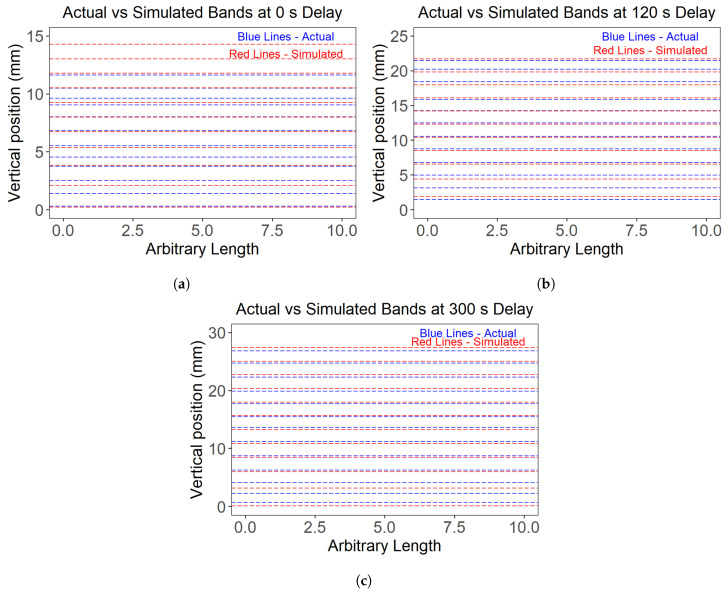
(**a**–**c**) The plots depicting the actual versus simulated white bands’ positions in the three wall structures. The actual band positions were measured from the center location of the wall with respect to the substrate surface, which is at a height of 0 mm. The simulated band positions were matched to the actual ones by optimizing the laser absorptivity into the thermal model.

The positions of the white bands were used as markers to validate the results of the thermal model. The laser absorptivity used as an input to the model was varied such that the simulated and actual white band positions matched. The fitted values of laser absorptivity for 0, 120, and 300 s interlayer delay were 0.37, 0.37, and 0.42 respectively. The actual band positions were measured from the optical micrographs of the walls. Furthermore, the band with its center located below the wall–substrate interface was not considered in the model prediction, as the model was designed to compute thermal histories only within the wall. Figure 16 shows the accuracy of the thermal model in terms of predicting the positions of the white bands in the three wall samples. The model does an excellent job of predicting the band positions for walls with extended delays. However, the accuracy is relatively inferior for the wall with a delay of 0 s. The 1D thermal model also appears to be more precise in predicting band positions away from the base plate where the heat dissipation becomes increasingly one-dimensional as opposed to the position of bands closer to the wall–base plate interface.

### 4.2. Process–Structure–Property Relationship

**Figure 17:**
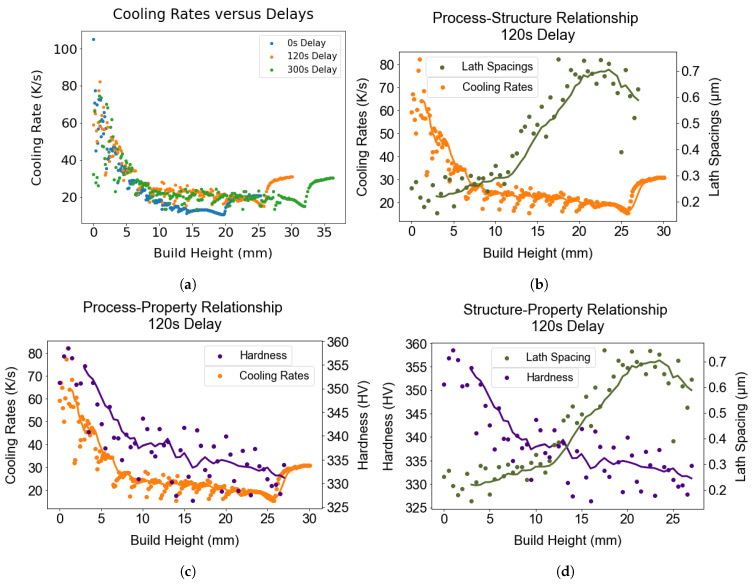
(**a**) Variation of cooling rates across the wall height is shown for three distinct interlayer delays. A pronounced decrease in cooling rates was observed up to a wall height of approximately 7.5 mm, regardless of the delay. Subsequently, the reduction of cooling rates slowed down with delays of 120 and 300 s, while they continued to decrease progressively with increasing height in the case of no delay. (**b**–**d**) Plots highlighting the relationship between cooling rates & microstructure, cooling rates & property, and microstructure & property for 120 s delay. These graphs demonstrate the process-structure-property relationship forged in this study. The solid lines in the figures indicate the moving average.

The process–structure–property relationship forged in the study can be expressed in terms of the correlation between cooling rates, lath thickness, and hardness. In this section, the relationship is discussed in detail only for the 120 s delay case for brevity. The thermal model used to estimate the cooling rates for the vertical positions in these wall components is shown in Figure 17a. It highlights that there is a trend of decreasing cooling rates with increasing wall height. Variations in alpha lath thickness and microhardness found along the wall height can be attributed to the variation in cooling rates with height in the wall specimens. The cooling rates gradually drop with increasing height as more layers are deposited on the wall components, corroborating the findings of systematic coarsening of the alpha laths with increasing build height, as shown in Figure 17c. Slower cooling allows sufficient time for alloying segregation, leading to coarsening of the lath spacing. In case of the shortest delay, the cooling rate drops continuously throughout the course of the build and attains the minimum value upon deposition of the last layer. The larger drop in cooling rates with height for the 0 s delay can be explained by the higher heat accumulation during the layer-by-layer deposition process. Adding delay between the deposition of subsequent layers tends to stabilize the cooling rates, and a trend of uniform cooling rates was observed above the third layer in wall structures built with an extended delay. The trend of hardness variations across the wall height is in good agreement with the cooling rates, as shown in Figure 17b. In Figure 17d, the variation in lath spacing captured along the wall height inversely correlates with the hardness variation with height, implying that there is a a Hall–Petch relationship between them. The gradual coarsening of the lath spacing observed with increasing build height results in a trend of reducing hardness, which highlights the structure–property relationship in the study.

## 5. Conclusions

In this present work, the effect of the interlayer delay period on the evolution of the thermal history, originating from heat accumulation, and its impact on the microstructure and properties of Ti-6Al-4V wall components fabricated using a wire-feed directed energy deposition technique was investigated. The results are summarized in Table 4. The results and analysis showed that the evolution of the melt pool dimensions, microstructures, and properties during DED processing is directly linked to the complex thermal history of the build and dynamic flow in a rapidly moving melt pool in the presence of layer-by-layer deposition. The conclusions of the study can be summarized as follows:

- The melt pool dimensions were directly influenced by the heat accumulation. It was observed that a delay of 300 s almost completely eliminated the heat buildup issues, leading to the stabilizations of the melt pool dimensions. A prolonged delay of 300 s decreased the variations in the melt pool length from 100.5% to 0.4% and the melt pool width from 14% to 1.5%, as compared to the 0 s delay condition. As a result, the printed wall component exhibited improved dimensional accuracy over the entire wall height.
- The positions of the white bands in the wall structures were a direct consequence of heat accumulation. The white bands were closely spaced in the case of the shortest delay due to the higher preheat temperature of the previously deposited layer. The white bands oriented perpendicular to the build direction suggest that there is a minimal thermal gradient across the thickness of the wall.
- The compositional analysis demonstrated that the aluminum weight percentage declines with increasing heat accumulation with wall height in the case of a 0 s delay, while the weight percentage of vanadium exhibited a minimal alteration in relation to interlayer delays. Because of the low confidence in the compositional data measured via the EDS technique, establishing a correlation between the variation in composition as a function of wall height with respect to interlayer delays was not achievable.
- The quantification of microstructural features using SEM images revealed that the alpha lath thickness progressively increases with increasing wall height, suggesting coarsening of the microstructure, due to reduced cooling rates with respect to the height. The average alpha lath spacing reduced from 0.49 to 0.23 μm with an increase to a 300 s interpass delay. The lath spacings were more consistent across the height up to 10 mm in the case of a longer delay, suggesting that a longer delay contributes to achieving relatively more homogeneous microstructures. The final microstructure was comprised of ≈90% alpha and ≈10% beta phases, and the phase composition remained similar irrespective of the interlayer delays.
- The Vickers microhardness and tensile testing results validated an improvement in the overall mechanical properties of the Ti64 wall structures with an increment of interlayer delay. The introduction of a 300 s delay led to an approximately 4% increase in hardness, with values rising from 334 HV to 346 HV. The yield stress increased from 817 MPa to 859 MPa, while the ultimate tensile stress increased from 914 MPa to 959 MPa with an extended delay of 120 s.

Therefore, the current investigation lays the groundwork for addressing the issues related to heat accumulation by implementing an optimized interpass delay in the laser hot wire DED process. The study also demonstrates how control of heat buildup can eliminate variabilities in the melt pool dimensions, promote greater uniformity in the microstructure along the height, and enhance the mechanical properties. The idea of incorporating interlayer delay in the LHW-DED process can be extended and applied to other wire-based direct energy deposition processes to address the challenges posed by heat accumulation.

## Figures and Tables

**Table 1:** The composition of the Ti-6Al-4V wire.

Elements	O	N	C	Fe	Al	V	Ti
Chemistry (wt%)	0.15	0.006	0.023	0.17	6.24	3.89	Balance

**Table 2:** Summary of the tensile properties of the three walls with distinct delays.

Properties	0 s Delay	120 s Delay	300 s Delay
Yield Stress (MPa)	817 ± 8.68	859.7 ± 9.17	825.3 ± 3.10
UTS (MPa)	914.9 ± 10.89	959 ± 5.31	927.5 ± 5.86
% Elongation	14.5 ± 5.17	7.5 ± 2.09	14.83 ± 1.33

**Table 3:** Mechanical properties of Ti-6Al-4V alloy for wrought samples and different wire-feed AM samples.

Properties	Wrought	WAAM	WLAM	WEBAM
Yield Strength (MPa)	948 [37] 880 [38] 760 [ASTM F136] 910–930 [AMS 4928]	856 ± 16 [39] 710 [40] 891 ± 9 [41] 712 [42]	825–835 [43]	846 [44] 956 [45]
Ultimate Tensile Strength (MPa)	994 [37] 950 [38] 825 [ASTM F136] 960–990 [AMS 4928]	993 ± 15 [39] 820 [40] 963 ± 8 [41] 872 [42]	905–915 [43] 1140 [46]	953 [44] 1020 [45]
Elongation (%)	21 [37] 14 [38] 8 [ASTM F136] 12–21 [AMS 4928]	17 ± 4 [39] 7.2 [40] 17.8 ± 0.6 [41] 11 [42]	11–12 [43] 6 [46]	4.5 [44] 8.3 [45]
Vickers Hardness (HV)	322 [ASTM F136]	332 [39]	332 ± 7 [46]	319 [45]

**Table 4:** Summary of results highlighting process–microstructure–property relationship forged in the study. The effect of increased interpass dwell time on the melt pool dimensions, heat accumulation, thermal histories, and resultant microstructures, as well as the properties of the as-deposited Ti64 wall specimens is summarized.

Properties		0 s Delay		120 s Delay		300 s Delay		Comments
Preheat Temp (C)	1st layer	214.6	207% ↑	225.6	39.3% ↑	242.3	8.5% ↑	delay ↑ heat buildup ↓
	15th layer	660.4		314.4		263.1		
Melt Pool Length (mm)	1st layer	15.3	100.5% ↑	15.6	58.8% ↑	23.15	0.4% ↓	delay ↑ instability ↓
	15th layer	30.6		24.7		23.06		
Melt Pool Width (mm)	1st layer	10.2	14% ↑	10.4	11.4% ↑	8.6	1.5% ↑	delay ↑ instability ↓
	15th layer	11.7		9.2		8.8		
Cooling Rates (K/s)	1st layer	80.3	79.6% ↓	86.2	65.8% ↓	86	57% ↓	delay ↑ drop of CR ↓
	15th layer	16.4		29.5		36.9		
Avg. Lath Spacing (μm)		0.49 ± 0.06		0.26 ± 0.04		0.23 ± 0.05		delay ↑ lath thickness ↓
Avg. Vickers Hardness (HV)		333.9 ± 7		336.9 ± 8		346.7 ± 8		delay ↑ hardness ↑
Avg. YS (MPa)		817 ± 8.7		859.7 ± 9.2		825.3 ± 3.1		delay ↑ YS ↑
Avg. UTS (MPa)		914.9 ± 10.9		959 ± 5.3		927.5 ± 5.86		delay ↑ UTS ↑

## Data Availability

The data presented in this study are available upon request from the corresponding author due to privacy restrictions.

## Abbreviations

The following abbreviations are used in this manuscript:

DED	directed energy deposition
SEM	scanning electron microscopy
EDS	energy dispersive spectroscopy
EDM	electrical discharge machining
